# Embodying Compassion: A Virtual Reality Paradigm for Overcoming Excessive Self-Criticism

**DOI:** 10.1371/journal.pone.0111933

**Published:** 2014-11-12

**Authors:** Caroline J. Falconer, Mel Slater, Aitor Rovira, John A. King, Paul Gilbert, Angus Antley, Chris R. Brewin

**Affiliations:** 1 Clinical Educational & Health Psychology, University College London, London, United Kingdom; 2 Department of Computer Science, University College London, London, United Kingdom; 3 Institució Catalana de Recerca i Estudis Avançats, University of Barcelona, Barcelona, Spain; 4 Mental Health Research Unit, University of Derby, Derby, United Kingdom; University of Bologna, Italy

## Abstract

Virtual reality has been successfully used to study and treat psychological disorders such as phobias and posttraumatic stress disorder but has rarely been applied to clinically-relevant emotions other than fear and anxiety. Self-criticism is a ubiquitous feature of psychopathology and can be treated by increasing levels of self-compassion. We exploited the known effects of identification with a virtual body to arrange for healthy female volunteers high in self-criticism to experience self-compassion from an embodied first-person perspective within immersive virtual reality. Whereas observation and practice of compassionate responses reduced self-criticism, the additional experience of embodiment also increased self-compassion and feelings of being safe. The results suggest potential new uses for immersive virtual reality in a range of clinical conditions.

## Introduction

Relating negatively to the self, in the form of excessive self-criticism, is one of the most significant psychological processes thought to influence the susceptibility to and maintenance of several psychopathologies [1], [2]. Positive self-relating such as self-compassion, on the other hand, is regarded as our natural regulator of mood and feelings of self-worth [3]–[5]. Recent approaches have focused on the use of imagery to facilitate and cultivate self-compassion in healthy and clinical populations, with promising results in improving well-being [6]–[8]. However, there are some who struggle with imagery, lacking any insightful impact from the experience, or who have their attempts undermined by lack of exposure to compassion, a fear of compassion, or a sense of not deserving compassion [9], [10].

In light of this, we investigated whether virtual reality (VR) could be a viable alternative to imagery-based approaches to enhance the experience of positive self-relating and self-compassion. At present, the focus of VR in mental health treatment has predominantly concerned enhancing existing interventions such as exposure for phobias and combat-related posttraumatic stress disorders, which has been very effective [11], [12]. Here we show how the emerging technique of virtual embodiment, leading to the illusion that a virtual body is attributed as the own body, may be used to inculcate feelings of self-compassion.

Research over the last two decades has shown that the brain is remarkably plastic with respect to body representation [13]. This field was inspired by the rubber hand illusion (RHI) [14] where it was shown that visual stimulation on a seen rubber hand placed in an anatomically plausible position on a table in front of a person, and temporally and spatially synchronous stimulation on the corresponding but hidden real hand, results in strong illusion that the rubber hand is their hand. This illusion has been shown to work well in immersive virtual reality, where instead of a rubber arm, a virtual arm is seen in stereo 3D as coming out of the real shoulder of the experimental participant [15].

Similar techniques have been used to induce body ownership with respect to a manikin body [16] and in immersive virtual reality it is possible to give people the illusion of ownership over a virtual body. They wear a head-tracked head-mounted display, and when they look down towards themselves they see a virtual body that is spatially coincident with their real body. Through real-time motion capture, when the person moves their real body they see the virtual body move correspondingly. They can also see this in a virtual mirror reflection (and shadows) of their body as well as looking directly towards it [16]. This is already a very powerful cue to the brain that this virtual body is their actual body. In our entire lives whenever we have looked down towards our body - or in a mirror - we have seen our body. Since the virtual reality is entirely programmed, the form or type of virtual body can be quite different to the person's real body. The brain seems to be remarkably adaptable in accepting alternative body representations, and in these circumstances generates the illusion that the virtual body is the own body. Of course at a cognitive level the participant knows that it is not their real body - but typically knowing that something is an illusion does not extinguish it. Hence the first person perspective (1PP) (the eyes of the virtual body coincident with the person's real eyes, and the virtual and real body being spatially coincident) is a very powerful factor towards inducing a whole body ownership illusion with respect to a virtual body [16], [17].

In such setups there is real-time motion capture of the participant's movements. Hence any movement of their real body can be mapped onto movements of the virtual body - to the extent supported by the motion capture. This visuomotor synchrony seems to be the second most powerful factor, after 1PP, in generating the whole body ownership illusion [18]. We use the term ‘embodiment’ to refer to the above setup - where a virtual body is spatially coincident with your real body, you see through the eyes of that virtual body, and with various degrees of synchronous multisensory correlation - such as visuomotor.

Recent evidence shows that these types of body illusions have physiological and psychological consequences: physiological changes due to the rubber hand illusion [19]; reductions in implicit racial bias in light-skinned people embodied in a dark-skinned virtual body [20]; and changes in size perception in bodies that look like children [21]. Hence, the meaning or semantics of the *type of body* seems to be carried with the illusion of ownership over that body. In our study we exploited the idea of embodiment in order to foster self-compassion, essentially by implicitly giving people the experience of delivering compassion to themselves.

In the first phase of our study highly self-critical participants interacted compassionately with a crying child avatar seated in front of them while embodied in an adult avatar. In the second phase, participants then embodied the child avatar and could re-experience their compassionate response from this embodied perspective. Our idea was that this would act as a very powerful mechanism for generating self-compassion; while embodied as the child participants would be on the receiving end of the compassion that they had themselves delivered. That is, while embodied as a child they are nevertheless still ‘themselves’ and therefore effectively they would have given themselves compassion. This mechanism is an objectification of the notion of self-compassion since the participants would have in fact given compassion to themselves. As a result of this, we hypothesized that levels of self-criticism would reduce and self-compassion would increase. Measures of positive and negative affect were also included as secondary variables to determine whether the intervention had specific effects on self-compassion or more global effects on mood.

As a control, a second group of participants viewed their compassionate response from a third person perspective. In other words they did not embody the child, but saw both the adult representation of themselves and the child from the outside. This condition allowed participants to re-experience their compassionate response but not from the embodied first-person perspective that was the target of their compassion. This condition also allowed us to control for any positive impact on self-relating that may have occurred through the act of being compassionate to another [22], [23].

## Methods

### Experimental Design and Participants

The design was between-groups comparing a first person perspective (1PP) and third person perspective (3PP) condition. By 1PP we mean that the participant saw the virtual body of the child egocentrically, from the viewpoint of the eyes of that body, and where the real and virtual body were spatially coincident. In the 3PP condition, people saw the scenario not embodied in the child body but from an external perspective. It has been shown that 3PP generally results in a lesser subjective illusion of ownership of the virtual body than 1PP. All participants while in the role of the adult were embodied with 1PP in the adult body.

As it has been hypothesized on the basis of theory that high levels of self-criticism should be a particular risk factor for depression in women [24], female undergraduate volunteers were recruited. Powering the design to detect a large effect size (Cohen's f  = 0.35, alpha  = 0.025, beta  = 0.2) indicated a sample size of 18 per group. We aimed to recruit up to an additional 6 participants per group to allow for technical failures and stopped when we had 24 participants in one group. Self-report of previous or current treatment for a mental health problem was an exclusion criterion, but no participants were excluded for this reason. Three participants were excluded in the 3PP version for technical reasons (the audio was not recorded so when embodied in the child they could not hear their previous compassionate comments spoken while in the role of the adult). Twenty-two females (mean age  = 22, *SD*  = 5.1), completed the first person perspective (1PP) condition and twenty-one females (mean age  = 22, *SD*  = 3.7) completed the third person perspective (3PP) condition. Participants were reimbursed for their time with either money or academic credit.

### Measures

#### Forms of Self-Criticizing/Attacking & Self-Reassuring Scale (FSCRS)

The FSCRS [25] was used to measure trait self-criticism and self-reassurance. Participants indicated on a 5-point Likert scale the extent to which various statements were true of themselves (0 =  not at all like me to 4 =  extremely like me). The scale comprises three subscales: inadequate self (IS, range 0–36; e.g. “There is a part of me that feels I am not good enough”), hated self (HS, range 0–20; e.g. “I stop caring about myself”), and reassured self (RS, range 0–32; e.g. “I find it easy to forgive myself”). The scale has high internal reliability, with Cronbach's alphas of.90 for IS, and.86 for HS and RS scales. The scale has been validated in both healthy and clinical populations [26].

#### Self-Compassion and Self-Criticism Scale (SCCS)

The SCCS (see Falconer et al., 2013, Manuscript S1) consists of five scenarios that are potentially self-threatening and can elicit varying degrees of self-criticism or self-compassion (e.g., “A third job rejection letter in a row arrives in the post”; “You arrive after walking to a meeting to find that you are late and the doors are closed”). Participants are instructed to imagine, as vividly as possible, that these scenarios are happening to them at the current moment in time and rate on 7-point Likert scales (1 =  not at all to 7 =  highly) the extent to which they would react to themselves in a Harsh, Contemptuous, Critical, Soothing, Reassuring, and Compassionate manner in relation to each imagined scenario. The scale is separated into two orthogonal subscales. The positive items are summed across scenarios to generate the Self-Compassion Scale (range 15–105) and the negative items are summed to generate the Self-Criticism Scale (range 15–105). The scale has good internal reliability with Cronbach's alphas of.91 and.87, respectively.

#### International Positive and Negative Affect Schedule, Short Form (I-PANAS-SF)

Positive affect (PA) and negative affect (NA) were measured with the 10-item I-PANAS-SF [27], a cross-culturally reliable and briefer version of the original PANAS [28]. Participants rated how strongly they were currently experiencing a particular emotion on a 5-point Likert scale (1 =  *not at all* to 5 =  *very much so*) (e.g. PA, range 5–25: active, inspired; NA, range 5–25: ashamed, hostile). Cronbach's alphas for the PA and NA scales are.78 and.76 respectively [27].

#### Two Forms of Positive Affect Scale (TFPAS)

The TFPAS measures the extent to which participants experience 18 different positive emotions [29] and has been used as a state scale of positive affect [30]. Factor analysis revealed three potential forms of positive affect: Active Affect (e.g. energetic, excited), Relaxed Affect (e.g. relaxed, calm) and Safe Affect (e.g. content, warm). The significance of this scale is that it allows for a better approximation of affect systems associated more specifically with self-compassion [4], [29]. Participants rate on a 5-point Likert scale (1 =  *not at all* to 5 =  *very much so*) how strongly they are experiencing these emotions at the current moment in time. There are 4 Safe Affect items (range 4–20), 6 Relaxed Affect items (range 6–30), and 8 Active Affect items (range 8–40). The authors reported a Cronbach's alpha of.83 for Active and Relaxed Affect, and.73 for Safe Affect.

#### Virtual Reality Experience Questionnaire

The purpose of this questionnaire (Information S1, Table 1) was to elicit the experience of presence in the virtual environment, the illusion of body ownership, and a set of questions regarding the specific experience. Presence is the illusion of being in the place depicted by the VR displays (Section A) and the question used is based on similar questions used in many past experiments [31]. The four illusion of body ownership questions and one illusion of agency question in relation to the adult (Section B), and the subset of these used in relation to the child (Section C) were based on previously used questions in earlier studies [20], [21] which were in turn based on the original RHI questionnaire [14]. The remainder of the questions were concerned with aspects of the actual experience.

**Table 1:** Mean (SD) scores on self-compassion, self-criticism, and mood pre- and post-intervention.

	1PP		3PP	
	Pre	Post	Pre	Post
Self-compassion	39.9 (12.6)	51.1 (13.7)	43.7 (13.8)	43.4 (16.1)
Self-criticism	67.2 (13.4)	55.5 (16.6)	60.0 (12.8)	53.5 (13.7)
I-PANAS-SF positive	15.8 (3.9)	16.0 (4.6)	15.5 (3.1)	15.1 (4.1)
I-PANAS-SF negative	7.8 (2.4)	7.0 (2.6)	8.2 (2.7)	7.6 (2.8)
Active Affect	17.8 (3.9)	16.5 (5.3)	16.8 (4.7)	17.5 (5.8)
Relaxed Affect	14.3 (3.3)	17.5 (3.2)	14.3 (3.9)	15.5 (4.8)
Safe Affect	11.5 (2.2)	13.2 (2.2)	11.3 (2.3)	11.3 (3.0)

1PP  =  1^st^ person perspective, 3PP  =  3^rd^ person perspective; Pre  =  pre-intervention, Post  =  post-intervention; I-PANAS-SF  =  Positive and Negative Affect Scales.

### Virtual Environment

The virtual room was created using Autodesk 3ds Max. It was an accurate 3D model of the actual room where the experiment was carried out. The furniture (curtain, door and stools) also matched the placement and size of them in the room. The only addition was a virtual mirror where the participants could see themselves in the virtual environment. The virtual characters used, both adult and child, were acquired from Rocketbox Studios. The scenario was implemented with Unity 3D 4 game engine.

The participant's head was tracked with a 6-DOF Intersense IS-900, a high-rate low-latency tracking system that allows the system to know the participant's head position and orientation and thus adjust the imagery to his perspective in real time. The rest of the body was tracked with Natural Point's Optitrack system with 37 light reflective passive markers attached to the participant's body. Twelve V100 infrared Optitrack cameras captured the tracking volume and body suits from Natural Point were used. To deliver the 3D imagery, we used an nVidia Quadro 4000 graphics card and an Oculus Rift (Developer Kit 1), a lightweight head-mounted display (HMD) that renders on a 1280×800 pixels resolution screen shared with both eyes and 90° minimum of horizontal field-of-view. The HMD is designed the way that each eye can only see half of the screen. On each frame, the scene is rendered twice, once on each half of the screen taking into account the disparity between the eyes' perspective.

### Ethics Statement

All procedures and materials were approved through the Ethics Committee of the University College London Division of Psychology and Language Sciences. Written informed consent was obtained.

### Procedure and Materials

Participants were recruited from the Psychology Department subject pool. They were first required to complete the FSCSR questionnaire online. If participants met the high self-criticism criterion (scoring in the upper third of a large sample of undergraduates, i.e. above 20, on the Inadequate Self subscale of the FSCSR) they were invited to a VR session. Upon arrival, and after written informed consent, participants completed the SSCCS, TPAS and the iPANAS, the presentation of which were randomized across participants.

Participants were then given information concerning compassion and task instructions that presented a staged approach to reducing distress based on current knowledge and practice in compassion focused therapy [4]. Participants were introduced to three essential stages for giving a compassionate response:

1. *Validation: The aim of this stage is to acknowledge that the other person is upset, that you do not judge them for this, and that it is perfectly acceptable for them to react in this way.*
2. *Redirection of Attention: The aim of this stage is to direct the other person's attention towards something that is more positive, soothing, and comforting.*
3. *Memory Activation: The aim of this stage is to suggest that the person could try to recall a memory of a person who loves or is kind to them. This memory is supposed to instil more positive feelings of warmth, comfort, and safety.*

Participants were also given generic sentences that corresponded to each of these three stages. They were instructed to memorize these sentences as best they could so as to deliver them slowly, softly, and compassionately to the child. Participants practiced their lines with the researcher to ensure that they were comfortable expressing themselves. After this, participants were fitted with the head mounted display (HMD) and body tracking suit (see Figure S1, panel A).

The first stage of the VR session (see Video S1) allowed participants to become accustomed to the virtual environment and their body. This was achieved through a series of guided exercises that involved standing and seated body movements while participants looked at themselves in a virtual mirror or looked down at their body. From earlier studies [20], [21], [32] we expected that the synchronous visual-motor feedback participants experienced during this time would lead to a strong sense of body ownership and agency with respect to their virtual body. Participants were also free to look around the virtual room, which was a virtual representation of the experiment room itself containing two stools. This stage lasted for approximately 3 minutes.

For the second stage of the experiment participants were seated on one of the stools opposite a seated crying child avatar (Figure S1, panel B). At their own pace they then began their compassionate response. The child was programmed to respond positively to the participants after the delivery of each stage of their response. For example, the child was initially hunched over and crying into their hands. This transitioned to the cessation of crying (although still sniffling) and a lowering of hands after the first stage of the compassionate response. The child then became less hunched over and stopped sniffling after the second stage of the compassionate response and then finally sat upright and elevated the head upon completion of the third stage of the compassionate response. The child movements and audio were previously recorded in conjunction with our three stage compassionate response using a female actor. After their compassionate response participants were asked to close their eyes to complete the body ownership illusion questions, which were recited to them and their responses were recorded by the researcher.

For the third stage of the experiment participants experienced a perspective change depending on their experimental condition. In the 1PP condition it was to be embodied in the body of the child (Figure S1, panel C), and in the 3PP condition facing both the child and adult bodies from the side of the room at a distance of 1 meter (Figure S1, panel D). In both conditions, participants were guided through the same exercises as before to become accustomed to their environment from this new perspective and, in the 1PP, to their new body. Participants in the 3PP condition did not have a virtual body. However, they could still freely look around the virtual environment and, to keep the conditions as similar as possible, they also completed the guided body movements.

For the fourth stage of the experiment participants re-experienced their compassionate response from their new perspective as the child. This included a real-time replay of their original adult avatar delivering compassion, which included the replay of their own body movements and voice. Participants were instructed to merely sit, look and listen to their compassionate response. Participants then exited the VR and completed the virtual reality experience questions including the body ownership illusion questions, and the SSCCS, TPAS and I-PANAS-SF. Participants were then asked to provide feedback on their experience, were fully debriefed and reimbursed for their participation.

## Results

Data are available in the file Data S1.

### Group Differences at Baseline

The two participant groups did not differ in initial trait levels of self-criticism on the FSCRS: Levels of inadequate self (mean ± *SD*: 26.1±4.6), *t*(41) = −.83, *p* = 0.41, reassured self (mean ± *SD*: 16.0±6.5,), *t*(41) = 1.02, *p* = 0.31, and hated self (mean ± *SD*:5.7±3.9), *t*(41) = −1.00, *p* = 0.32.

### Presence, Body Ownership and Agency

The questions relating to presence, body ownership and agency are given in Information S1. Table S1 within Information S1 gives the median and interquartile ranges for these questions, and the results are in line with those observed in previous studies, for example, Banakou et al. (2013). Table S2 within Information S1 indicates that during the first phase of the experiment (embodiment in the adult avatar), prior to the group manipulation, the 1PP group showed some evidence of greater body ownership and agency than the 3PP group, even though their experience to that point was identical. We therefore took account of these differences in subsequent analyses.

### Self-Compassion and Self-Criticism

Self-criticism and self-compassion scores before and after the VR session were analysed in two separate mixed-model 2×2 analyses of variance whereby Time (pre- and post-intervention) was the within-subjects variable and Perspective (1PP, 3PP) was the between-subjects variable. The data are summarized in Table 1.

For self-compassion, there was no effect of Perspective, *F*(1,41) = .23, *p* = .64, but there was a significant main effect of Time, *F*(1,41) = 14.33, *p*<.001, *η*_p_^2^ = .26, qualified by a significant interaction between Time and Perspective, *F*(1,41) = 15.87, *p<*.001, *η*_p_^2^ = .28. As indicated in Table 1, in the 3PP condition there is no change from pre- to post-, but there is a very strong increase in compassion in the 1PP condition. Multiple comparisons (Duncan method) show that for 1PP the difference post-pre has a 95% confidence interval between 6.9 and 15.5, whereas the confidence interval for 3PP includes 0 (−4.7 to 4.1).

In order to control for baseline differences in overall body ownership and agency we computed a summary body ownership variable by summing the relevant four items. This internally reliable measure (coefficient alpha  = 0.86), as well as the single body agency item, were then used as covariates to rule out the possibility that these baseline differences could account for our results. After controlling for initial levels of body ownership and agency relative to the adult avatar, the Time × Perspective interaction was still highly significant (*p*<.001).

For self-criticism there was no effect of Perspective, F(1,41) = 1.27, p = .266, but a large effect of Time, *F*(1,41) = 42.1, *p<*.001, *η*_p_^2^ = .51. There was a moderate size interaction between Time and Perspective that fell short of significance, *F*(1,41) = 3.46, *p = *.07, *η*_p_^2^ = .08. The data indicate that after re-experiencing their compassionate response participants decreased their self-criticism level, irrespective of whether they viewed their response from a 1PP or 3PP perspective. Controlling for initial levels of body ownership and agency relative to the adult avatar did not change these results.

### Mood

Positive and negative affect as measured by the I-PANAS-SF were not responsive to the experimental scenario, as indicated by the lack of any significant effects of Perspective, Time, or Time × Perspective interactions. The largest effect was a main effect of Time on negative affect, *F*(1,41) = 3.62, *p = *.06, *η*_p_^2^ = .08. Equally, for active affect as measured by the TFPAS there were no significant main effects or interaction, largest F(1, 41) = 2.61, p = .11. Controlling for initial level of body ownership and agency relative to the adult avatar did not change these results. For relaxed affect there was a main effect of Time, *F*(1,41) = 12.55, *p = *.001, *η*_p_^2^ = .23, such that participants became more relaxed post-intervention, but no significant effect of Perspective or interaction, largest *F*(1,41) = 2.67, *p = *.11. Controlling for initial level of body ownership relative to the adult avatar did not change these results, but controlling for agency abolished the significant effect of Time. This appeared to be due to a nonsignificant positive correlation between agency and change in relaxed affect over time.

For safe affect there was no significant effect of Perspective, F(1,41) = 2.31, p = .136, but there was a significant effect of Time, *F*(1,41) = 5.45, *p = *.025, *η*_p_^2^ = .12, and a significant Time × Perspective interaction, *F*(1,41) = 5.45, *p = *.025, *η*_p_^2^ = .12. Multiple comparisons with Duncan's corrections showed that the only group difference where the 95% confidence interval does not include zero is for the 1PP condition (the confidence interval of the difference post- mean – pre-mean is 0.66 to 2.70). Thus the 1PP condition was the only one to be associated with an increase in safe affect over time. After controlling for initial level of body ownership relative to the adult avatar, both the main effect of Time and the interaction remained significant (*p*<.05). After controlling for agency the interaction remained significant (*p*<.05) but the Time effect was no longer significant. This appeared to be due to a nonsignificant positive correlation between agency and change in safe affect over time.

## Discussion

The key finding from this study is that while simply rehearsing the delivery and receipt of compassionate behavior leads to a reduction in self-criticism, exploiting the IVR technique of embodiment in the recipient has the additional effect of positively increasing self-compassion in naturally self-critical individuals. This is one of the first demonstrations that immersive virtual reality can be used to inculcate positive emotions that are important for mental health as well as decrease negative emotions such as fear.

From our data we cannot rule out the possibility that embodiment in the recipient could also have an additional effect on lessening self-criticism, as such an effect might have been detectable with a larger sample. At this stage the safest conclusion is that effects appear to be strongest for compassion. These results are consistent with previous findings suggesting that these two ways of self-relating are not opposite sides of the same coin but can be experienced orthogonally (see Falconer et al., 2013, Manuscript S1). That is, low levels of self-criticism are not synonymous with high levels of self-compassion. Importantly, our data also showed that the effect was not mirrored by a general change in positive and negative affect. Moreover, the degree of increase in self-compassion or decrease in self-criticism were not significantly correlated with changes in either positive or negative mood. This suggests that although embodiment may impact mood such effects are unlikely to be responsible for our findings.

One possible caveat is the finding that those in the 3PP condition tended to have lower body ownership and agency than those in the 1PP condition, even though these questions referred to experiences prior to the experimental manipulation that were identical in the two groups. Analyses showed that although those who had the stronger illusions with respect to these variables and presence were precisely those who showed the greater increase in self-compassion comparing Pre to Post, these differences were not responsible for our major findings, which were present whether or not we controlled for initial body ownership and agency.

Re-experiencing one's own compassion in the egocentric 1PP setup also increased feelings of safe affect, which is the type of positive affect most closely associated with affiliative emotions and encounters, and is thought to arise from a soothing-contentment affect regulation system [29], [33]. We hypothesized that embodying the child would create a situation in which the participant can passively experience self-compassion and its affiliative emotions. That is, embodying the child avatar, while retaining a self-identification with the original adult avatar during the compassionate response, would elicit a self-to-self situation through which participants can experience simulated self-compassion. It is important to note, however, that in the scenario there was no direct intention to be compassionate towards the self, rather the intention was experienced indirectly. It appears that the sensory experience of hearing one's own compassionate words spoken from a first person perspective was sufficient to increase self-compassion. This is consistent with studies reviewed in the introduction, in which embodiment effects were shown to include largely automatic physiological and psychological changes. More specifically, first-person memories of receiving compassion from the self within the experiment may have been more salient or more emotionally-laden than third-person memories when it came to completing the measure of self-compassion.

The exact mechanism or mechanisms responsible cannot be determined from this study. They may be to do either with the nature of the virtual body, or with the nature of the interaction with that body, or both. Previous research has demonstrated effects attributable to the semantics of the specific virtual body that is owned, for example when adoption of a virtual child body leads to unconscious changes in size perception. Although we have ruled out any influence of degree of embodiment in the adult avatar, we have not shown that embodiment in the child is the active ingredient, or indeed is necessary. It is possible that the adoption of other virtual bodies would be equally effective, and this remains to be determined. There are also important questions about the active ingredient in the interaction with the virtual body. Taking a first rather than third person perspective has itself been linked with increased access to emotion and body state information [34], and our effects may be due to perspective-taking, embodiment, or a combination of the two.

There was some reason to anticipate that participants in the 3PP condition would have experienced a negative impact on self-relating. Replaying autobiographical events from a 3PP evokes an evaluative assessment of behavior [35], and more specifically negative self–evaluation in the case of depressed patients [36]. However, participants in 3PP, as well as those in the 1PP, exhibited a strong decrease in self-criticism levels. Although this may be attributed to regression to the mean, participants in both conditions also showed an increase in relaxed affect, which is a form of positive affect thought to arise from the same soothing-contentment affect regulation system as safe affect [29]. Our results are consistent with previous findings that show compassion for others is associated with a reduction in depressive symptoms and a more favorable self-view [22], [23]. These positive changes may have overridden any negative self-evaluations that could have arisen as a result of the 3PP view.

Self-to-self simulations such as we employed in this study are not uncommon in psychotherapy. Two-chair interventions, for example, based on Gestalt therapy principles [37], have been used with some success to resolve conflict between the “critical self” and the “experiencing self” [38]. Similarly, compassion-focused therapy [4], grounded in an evolutionary approach to depression, endeavors to alter critical self-to-self relations through the use of compassionate imagery aimed at soothing and reassuring the “experiencing self” [6]. However, clinical reports indicate that some individuals struggle to express self-compassion [10], [38]. One potential advantage of the current paradigm over existing methods is that experiencing self-compassion is achieved indirectly. This may assist in overcoming some of the resistance that is thought to arise as a result of individuals feeling undeserving of compassion. Furthermore, the procedure reminds participants that self-compassion is analogous to compassion for others, a view which is often neglected in Western society [39].

In other research in our laboratory we are testing the proposition that embodiment leads to stronger changes in self-compassion than first-person imagery employed without virtual reality, and investigating whether repeated sessions lead to deeper or longer-lasting changes in self-compassion. It will also be important to test the paradigm with male samples and within clinical populations to determine whether embodiment processes in VR can be applied as a tool for the treatment of maladaptive self-relating in psychopathology.

## Supporting Information

Figure S1 **Virtual environment used in the experiment: (A) suit and and head-mounted display; (B) view of child avatar when embodied in adult avatar; (C) view of adult avatar when embodied in the child avatar (1^st^ person perspective); (D) external view of child and adult avatars (3^rd^ person perspective).**
(EPS)

Manuscript S1 Falconer et al. (2013) Demonstrating Mood Repair with a Situation-Based Measure of Self-Compassion and Self-Criticism.
(DOCX)

Information S1 Methodological Details.
(DOCX)

Data S1 (SAV)

Video S1 (ZIP)

## References

[1] Hewitt PL, Flett GL (2002) Perfectionism and stress processes in psychopathology. In: Flett GL, Hewitt PL, editors. Perfectionism: Theory, research, and treatment. Washington, D.C.: American Psychological Association. pp.255–284.

[2] Blatt SJ, Zuroff DC (1992) Interpersonal relatedness and self-definition - 2 prototypes for depression. Clin Psychol Rev 12: 527–562.

[3] Neff KD, Vonk R (2009) Self-compassion versus global self-esteem: two different ways of relating to oneself. J Pers 77: 23–50. 19076996 10.1111/j.1467-6494.2008.00537.x

[4] Gilbert P (2010) Compassion focused therapy: Distinctive features. London: Routledge.

[5] Leary MR, Tate EB, Adams CE, Allen AB, Hancock J (2007) Self-compassion and reactions to unpleasant self-relevant events: the implications of treating oneself kindly. J Pers Soc Psychol 92: 887–904. 17484611 10.1037/0022-3514.92.5.887

[6] Gilbert P, Irons C (2004) A pilot exploration of the use of compassionate images in a group of self-critical people. Memory 12: 507–516. 15487546 10.1080/09658210444000115

[7] Neff KD, Germer CK (2013) A pilot study and randomized controlled trial of the mindful self-compassion program. J Clin Psychol 69: 28–44. 23070875 10.1002/jclp.21923

[8] Hofmann SG, Grossman P, Hinton DE (2011) Loving-kindness and compassion meditation: Potential for psychological interventions. Clinical Psychology Review 31: 1126–1132. 21840289 10.1016/j.cpr.2011.07.003 PMC3176989

[9] Gilbert P, McEwan K, Matos M, Rivis A (2011) Fears of compassion: Development of three self-report measures. Psychol Psychother 84: 239–255. 22903867 10.1348/147608310X526511

[10] Gilbert P, McEwan K, Catarino F, Baião R, Palmeira L (2013) Fears of happiness and compassion in relationship with depression, alexithymia, and attachment security in a depressed sample. Brit J Clin Psychol doi:101111/bjc12037 10.1111/bjc.12037 24283291

[11] Pan X, Gillies M, Barker C, Clark DM, Slater M (2012) Socially anxious and confident men interact with a forward virtual woman: an experimental study. PLoS One 7: e32931. 22509251 10.1371/journal.pone.0032931 PMC3324473

[12] Gerardi M, Cukor J, Difede J, Rizzo A, Rothbaum B (2010) Virtual reality exposure therapy for post-traumatic stress disorder and other anxiety disorders. Curr Psychiatry Rep 12: 298–305. 20535592 10.1007/s11920-010-0128-4

[13] Blanke O (2012) Multisensory brain mechanisms of bodily self-consciousness. Nat Rev Neurosci 13: 556–571. 22805909 10.1038/nrn3292

[14] Botvinick M, Cohen J (1998) Rubber hands 'feel' touch that eyes see. Nature 391: 756–756. 9486643 10.1038/35784

[15] Slater M, Perez-Marcos D, Ehrsson HH, Sanchez-Vives MV (2008) Towards a digital body: the virtual arm illusion. Front Hum Neurosci 2 10.3389/Neuro.3309.3006.3200 PMC2572198 18958207

[16] Petkova VI, Ehrsson HH (2008) If I were you: Perceptual illusion of body swapping. PLoS One 3 10.1371/journal.pone.0003832 PMC2585011 19050755

[17] Slater M, Spanlang B, Sanchez-Vives MV, Blanke O (2010) First person experience of body transfer in virtual reality. PLoS One 5: e10564. 20485681 10.1371/journal.pone.0010564 PMC2868878

[18] Kokkinara E, Slater M (2014) Measuring the effects through time of the influence of visuomotor and visuotactile synchronous stimulation on a virtual body ownership illusion. Perception 43: 43–58. 24689131 10.1068/p7545

[19] Barnsley N, McAuley JH, Mohan R, Dey A, Thomas P, et al (2011) The rubber hand illusion increases histamine reactivity in the real arm. Curr Biol 21: R945–R946. 22153159 10.1016/j.cub.2011.10.039

[20] Peck TC, Seinfeld S, Aglioti SM, Slater M (2013) Putting yourself in the skin of a black avatar reduces implicit racial bias. Consciousness Cogn 22: 779–787. 10.1016/j.concog.2013.04.016 23727712

[21] Banakou D, Groten R, Slater M (2013) Illusory ownership of a virtual child body causes overestimation of object sizes and implicit attitude changes. Proc Natl Acad Sci USA 110: 12846–12851. 23858436 10.1073/pnas.1306779110 PMC3732927

[22] Mongrain M, Chin JM, Shapira LB (2011) Practicing compassion increases happiness and self-esteem. J Happiness Stud 12: 963–981.

[23] Alden LE, Trew JL (2013) If it makes you happy: Engaging in kind acts increases positive affect in socially anxious individuals. Emotion 13: 64–75. 22642341 10.1037/a0027761

[24] Blatt SJ (2004) Experiences of depression: Theoretical, clinical and research perspectives. Washington, D.C.: American Psychological Association.

[25] Gilbert P, Clarke M, Hempel S, Miles JN, Irons C (2004) Criticizing and reassuring oneself: An exploration of forms, styles and reasons in female students. Br J Clin Psychol 43: 31–50. 15005905 10.1348/014466504772812959

[26] Castilho P, Pinto-Gouveia J, Duarte J (2013) Exploring self-criticism: Confirmatory factor analysis of the FSCRS in clinical and nonclinical samples. Clin Psychol Psychother doi:101002/cpp1881 10.1002/cpp.1881 24307461

[27] Thompson ER (2007) Development and validation of an internationally reliable short-form of the Positive and Negative Affect Schedule (PANAS). Journal of Cross-Cultural Psychology 38: 227–242.

[28] Watson D, Clark LA, Tellegen A (1988) Development and validation of brief measures of positive and negative affect - The PANAS scales. J Pers Soc Psychol 54: 1063–1070. 3397865 10.1037//0022-3514.54.6.1063

[29] Gilbert P, McEwan K, Mitra R, Franks L, Richter A, et al (2008) Feeling safe and content: A specific affect regulation system? Relationship to depression, anxiety, stress, and self-criticism. J Posit Psychol 3: 182–191.

[30] Rockliff H, Karl A, McEwan K, Gilbert J, Matos M, et al (2011) Effects of intranasal oxytocin on 'compassion focused imagery'. Emotion 11: 1388–1396. 21707149 10.1037/a0023861

[31] Sanchez-Vives MV, Slater M (2005) From presence to consciousness through virtual reality. Nat Rev Neurosci 6: 332–339. 15803164 10.1038/nrn1651

[32] Kilteni K, Bergstrom I, Slater M (2013) Drumming in immersive virtual reality: the body shapes the way we play. IEEE Trans Vis Comput Graph 19: 597–605. 23428444 10.1109/TVCG.2013.29

[33] Depue RA, Morrone-Strupinsky JV (2005) A neurobehavioral model of affiliative bonding: implications for conceptualizing a human trait of affiliation. Behav Brain Sci 28: 313–350. 16209725 10.1017/S0140525X05000063

[34] Brewin CR, Gregory JD, Lipton M, Burgess N (2010) Intrusive images in psychological disorders: characteristics, neural mechanisms, and treatment implications. Psychol Rev 117: 210–232. 20063969 10.1037/a0018113 PMC2834572

[35] Libby LK, Eibach RP, Gilovich T (2005) Here's looking at me: The effect of memory perspective on assessments of personal change. J Pers Soc Psychol 88: 50–62. 15631574 10.1037/0022-3514.88.1.50

[36] Kuyken W, Moulds ML (2009) Remembering as an observer: How is autobiographical memory retrieval vantage perspective linked to depression? Memory 17: 624–634. 19536690 10.1080/09658210902984526

[37] Wagner-Moore LE (2004) Gestalt therapy: Past, present, theory, and research. Psychotherapy 41: 180–189.

[38] Shahar B, Carlin ER, Engle DE, Hegde J, Szepsenwol O, et al (2012) A pilot investigation of emotion-focused two-chair dialogue intervention for self-criticism. Clinical Psychology & Psychotherapy 19: 496–507. 21710579 10.1002/cpp.762

[39] Goetz JL, Keltner D, Simon-Thomas E (2010) Compassion: An evolutionary analysis and empirical review. Psychol Bull 136: 351–374. 20438142 10.1037/a0018807 PMC2864937

