# Supplementary material for: Embodying Compassion: A Virtual Reality Paradigm for Overcoming Excessive Self-Criticism
Source: PLoS One. 2014 Nov 12;9(11):e111933. doi: 10.1371/journal.pone.0111933 (PMC4229123; doi:10.1371/journal.pone.0111933)
Supplement: Manuscript S1 — Falconer et al. (2013) Demonstrating Mood Repair with a Situation-Based Measure of Self-Compassion and Self-Criticism. (DOCX) [file pone.0111933.s002.docx]

**Demonstrating Mood Repair with a Situation-Based Measure of Self-Compassion and Self-Criticism**

Caroline J. Falconer^1*,^ John A. King^1^ and Chris R. Brewin^1^

*^1^Department of Clinical, Educational & Health Psychology, University College London, London, UK.*

***Correspondence to:**

Dr Caroline J. Falconer

Department of Clinical, Educational & Health Psychology,

University College London,

Gower Street, London

WC1E 6BT,

U.K.

Tel: +44 (0)207 679 1826

Fax: +44 (0)207 619 1989

Email: [c.falconer@ucl.ac.uk](mailto:c.falconer@ucl.ac.uk)

Word Count: 5,495

**Abstract**

The clinical significance of self-criticism and self-compassion has prompted the development of measures assessing these constructs. However, there is a lack of measures assessing their interaction within specific contexts and potential involvement in mood repair processes. To rectify this we developed the Self-Compassion and Self-Criticism Scale (SCCS). In Study 1 Exploratory Factor Analysis (n=413) showed a clear two-factor structure of the SCCS denoting two orthogonal scales, with high internal validity (α ≥ .87). Correlations between the SCCS and existing measures also demonstrated appropriate convergent validity. Study 2 (n=90) provides preliminary evidence that the SCCS can detect changes in self-appraisals. Participants receiving no performance feedback from a difficult language task showed reduced state self-criticism and increased state self-compassion, demonstrating mood repair.

**Key Words:** Self-Compassion, Self-Criticism, Feedback, Mood Repair.

**Demonstrating Mood Repair with a Situation-Based Measure of Self-Compassion and Self-Criticism**

Adding to a long-standing interest in self-criticism there has been an increased focus over the last decade in measuring self-compassion, and several reliable and valid questionnaires of both constructs now exist ([Blatt, D'Afflitti, & Quinlan, 1979](#_ENREF_4); [Brewin & Shapiro, 1984](#_ENREF_7); [Gilbert, Clarke, Hempel, Miles, & Irons, 2004](#_ENREF_11); [Neff, 2003b](#_ENREF_24)). However, there is a lack of research addressing how processes of self-compassion and self-criticism interact at specific moments in time or under specific circumstances. Coinciding with this gap in the research is an absence of measures that assess both self-compassion and self-criticism in parallel. The current study sought to develop and validate a new measure incorporating both constructs, with the ultimate aim of furthering understanding of the dynamic relationships between them.

Excessive self-criticism is one of the most significant psychological processes thought to influence the susceptibility to and the maintenance and relapse of many psychopathologies ([Brewin & Firth-Cozens, 1997](#_ENREF_5); [Hewitt & Flett, 2002](#_ENREF_15); [Ingram, 2003](#_ENREF_16); [Koerner & Linehan, 1996](#_ENREF_17); [Pagura, Cox, Sareen, & Enns, 2006](#_ENREF_31); [Southwick, Yehuda, & Giller, 1995](#_ENREF_34)). Maladaptive self-criticism can be defined as a persistent tendency for negative self-evaluation that instils feelings of shame and low self-worth. The aetiology of chronic self-criticism is thought to arise early in life through a lack of or deficient affiliative relationships ([Andrews, 1995](#_ENREF_2); [Andrews, Brewin, Rose, & Kirk, 2000](#_ENREF_3); [Brewin, Firth-Cozens, Furnham, & McManus, 1992](#_ENREF_6); [Koestner, Zuroff, & Powers, 1991](#_ENREF_18); [Sachs-Ericsson, Verona, Joiner, & Preacher, 2006](#_ENREF_33)).

Equally important for psychopathology is a deficit in the ability to self-soothe and reassure, which is also thought to arise from inadequate nurturing during childhood ([Gilbert, 2010b](#_ENREF_10)) and can amplify negative self-appraisals ([Gilbert, 2010a](#_ENREF_9); [Gilbert, 2010b](#_ENREF_10)). Compassionate self-soothing and reassurance has been regarded as our natural regulator of shame and self-criticism ([Gilbert, 2010a](#_ENREF_9); [Leary, Tate, Adams, Allen, & Hancock, 2007](#_ENREF_20); [Neff, 2003b](#_ENREF_24); [Neff & Vonk, 2009](#_ENREF_30)). Indeed, self-compassion is a good predictor of positive affect and happiness, is associated with more enduring feelings of self-worth ([Neff & Vonk, 2009](#_ENREF_30)), and predicts coping in the face of failure and stress ([Neely, Schallert, Mohammed, Roberts, & Chen, 2009](#_ENREF_22); [Neff, Hsieh, & Dejitterat, 2005](#_ENREF_26)). Developing and enhancing self-compassion through training has significantly reduced self-criticism, shame and depression in chronically depressed patients ([Gilbert & Irons, 2004](#_ENREF_12); [Gilbert & Procter, 2006](#_ENREF_14)) and has even improved psychological wellbeing in healthy individuals ([Leary et al., 2007](#_ENREF_20); [Neff & Germer, 2012](#_ENREF_25)).

To our knowledge there have been very few studies investigating self-criticism and self-compassion simultaneously. One exception was [Longe et al. (2010](#_ENREF_21)), who investigated the neural correlates underlying self-criticism and self-compassion in an fMRI study. Participants were asked to imagine themselves in various scenarios that could be potentially self-threatening (i.e. receiving a job rejection letter). While imagining themselves in each scenario they were asked to react either critically or reassuringly towards themself. Self-critical responses activated the prefrontal cortex and the dorsal anterior cingulate cortex while self-reassuring responses resulted in activation of the insula. However, despite these important findings [Longe et al. (2010](#_ENREF_21)) did not assess the subjective responses to each of the scenarios.

Longe et al.’s (2010) data suggest self-criticism and self-compassion are, at least partially, independent processes. Future studies would benefit from a standardised questionnaire measurement that included both constructs, in order to investigate short-term dynamic interactions between them and their associations with changes in mood. To this end the Self-Compassion and Self-Criticism Scale (SCCS) was developed to measure compassionate and critical self-appraisals at a specific moment in time.

**1.2 Overview of SCCS Development**

In our view self-compassion and self-criticism are complex emotional responses that have specific temporal relationships and may be difficult for individuals to assess in the abstract. For this reason, we developed the SCCS around imagined scenarios, inspired by [Longe et al. (2010](#_ENREF_21)). The initial version of the scale consisted of eight scenarios that participants can respond to with varying degrees of self-criticism or self-compassion. Participants are instructed to imagine, as vividly as possible, that these scenarios are happening to them at the current moment in time. Participants are required to rate on a 7-point Likert scale (*1=not at all to 7=highly*) the extent to which they would react to themselves in different ways, in response to each imagined scenario. Initially each scenario had four self-compassionate and five self-critical reactions. Self-compassion was operationalized as self-kindness or self-reassurance, consistent with Gilbert (2010a, b). Scenarios and responses were established through discussions with colleagues in the clinical psychology field.

In study one the SCCS was pilot tested in a group of 413 participants and exploratory factor analysis (EFA) was used to examine the factor structure and internal validity of responses within and across the scenarios. Based on the results of this study we reduced the length of the scale by removing three scenarios and three response items. The relationships between the SCCS and existing measures of self-criticism and self-compassion were then investigated. Previous research has suggested both that the two constructs are independent with moderate negative correlations between the two ([Gilbert et al., 2004](#_ENREF_11); [Neff, 2003b](#_ENREF_24)), or that they form two poles of a single dimension ([Neff, 2003b](#_ENREF_24)). In study two we tested the sensitivity of the SCCS to experimental manipulations and its relation to changes in different types of affect. The measure was administered to a group of 90 students before and after a difficult language task. Scores were analysed in relation to the different feedback participants received after the task.

**2.1. Study One Method**

**2.1.1. Participants.** A total of 413 participants took part in this study (254 females; 159 males). The majority of participants ranged between the ages of 18 – 24 years old (62.6%) followed by 19.9% ranging between 25-34 years old, 10.2% ranging between 45-54 years old, 5.1% ranging between 35-44 years old, and 2.2% ranging between 55-64 years old. Experimental procedures were approved by University College London’s Psychology and Language Sciences Ethics Committee.

**2.1.2. Procedure.** Participants were recruited through a University College London online survey that was advertised throughout the university and on social networking sites. Participants were given a URL link to the online survey and provided informed consent to participate. Demographic information was recorded including age range and sex. In addition to the SCCS, participants completed several other questionnaires assessing levels of self-compassion and self-criticism, which are outlined below. Debriefing information was provided at the end of the study. Questionnaire presentation and data collection were accomplished using the Qualtrics online survey platform and the order of questionnaire presentation was randomised across participants.

**2.1.3. Measurements.**

*Self-Compassion and Self-Criticism Scale (SCCS)*

The initial version of the SCCS consisted of eight scenarios that are potentially self-threatening and can elicit varying degrees of self-criticism or self-compassion:

1. A third job rejection letter in a row arrives in the post.

2. You arrive after walking to a meeting to find that you are late and the doors are closed.

3. You arrive home to find that you have left your keys at work.

4. You receive a letter in the post that is an unpaid bill reminder.

5. You have just dropped and scratched your new Smart phone.

6. You have just received a failed test result.

7. You have just opened the washing machine door to find that your white wash has turned pink.

8. After searching your bag you realize that you have lost a £20 note.

Participants are required to rate on 7-point Likert scales (*1=not at all* to *7=highly*) the extent to which they would react to themselves in a Harsh, Contemptuous, Hostile, Cold, Critical, Soothing, Reassuring, Compassionate and Warm manner in relation to each imagined scenario.

*Forms of Self-Criticizing/Attacking & Self-Reassuring Scale (FSCRS)*

The FSCRS ([Gilbert et al., 2004](#_ENREF_11)) is a measure of self-criticism and self-reassurance. Participants respond on a 5-point Likert scale the extent to which various statements are true of them (1=*not at all like me* to 5=*extremely like me*). The scale comprises three subscales: inadequate self (IS: e.g., “I am easily disappointed”), hated self (HS: e.g., “I stop caring about myself”), and reassured self (RS: e.g., “I find it easy to forgive myself”). The authors reported Cronbach’s alphas of .90 for the IS, and .86 for the HS and RS scales.

*Self-Criticism Scale (SC)*

This scale was developed by [Brewin et al. (1992](#_ENREF_6)) and is a combination of items from the Depressive Experiences Questionnaire ([Blatt et al., 1979](#_ENREF_4)) and the scale of Responsibility for Negative Outcomes ([Brewin & Shapiro, 1984](#_ENREF_7)), both of which have acceptable reliability and validity. This 9-item scale is concerned with self-criticism and the perception of blameworthiness regarding life outcomes. Participants rate on a 7 point Likert scale the extent to which they agree or disagree (1= Strongly Disagree to 7 = Strongly Agree) with statements of self-criticism (“I often find that I don’t live up to my own standards or ideals”) and self-blame (“My misfortunes have resulted mainly from the mistakes I’ve made”). The authors reported a Cronbach’s alpha of .83.

*Self-Compassion Scale (SCS)*

The SCS ([Neff, 2003b](#_ENREF_24)) measures six aspects of self-compassion and includes 26items rated on a 5-point Likert scale of frequency (1=*almost never* to 5=*almost always*). The six subscales are: self-kindness, self-judgment, common humanity, isolation, mindfulness, and over-identification. The author reported Cronbach’s alphas of .75 - .81 for subscales and .92 for the whole scale. In view of recent evidence that the subscales are independent and do not measure a single overarching compassion construct ([Williams, Dalgleish, Karl, & Kuyken, 2014](#_ENREF_37)), the current study utilized the self-kindness subscale as the closest conceptually to the SCCS (e.g. “I’m kind to myself when I’m experiencing suffering”).

**3.1 Results**

**3.1.1. Exploratory Factor Analysis.** We first conducted an EFA of the response items for each of the eight scenarios to examine the factor structure and to establish item retention. A principal components method^[[1]](#footnote-1)^ with direct oblimin rotation was used to allow any factors to correlate.

Each scenario EFA showed a strong Kaiser-Meyer-Olkin measure of sampling adequacy (> .76) and a significant Bartlett’s test of sphericity (*p* < 0.001). A two factor structure was revealed for each scenario, as indicated by Eigenvalues greater than one. The first factor of each scenario included all of the self-critical items with factor loadings ranging from 0.67 to 0.89. The percentage of explained variance from this factor ranged between 33.3% - 40.3% across scenarios. The second factor for each scenario was comprised of the self-compassionate items with factor loadings ranging from 0.72 to 0.92. The percentage of explained variance from this factor ranged between 23.3% - 33.3% across scenarios. The combined explained variance of both factors ranged between 63% - 70% across scenarios. With the exception of scenario one (*r* = -0.23), the two factors did not significantly correlate with one another (*p* > 0.05). Summaries of these results are available on request.

Two additional EFAs were conducted to establish the nature of any inherent factor structure among the scenarios. The scenarios were analysed with separate summed whole scores from the self-compassion and self-criticism factors established in the first EFA. The EFA showed a strong Kaiser-Meyer-Olkin measure of sampling adequacy (.94) and a significant Bartlett’s test of sphericity (*p* < 0.001) for the self-compassion factor across scenarios. The self-criticism factor also had a strong Kaiser-Meyer-Olkin measure of sampling adequacy (.91) and a significant Bartlett’s test of sphericity (*p* < 0.001). The EFA revealed a one factor structure from the scenarios for both self-compassion and self-criticism responses. The factor loadings ranged from .68 to .85 for the self-compassion responses accounting for 66% of the explained variance. Factor loadings for the self-criticism responses ranged from .75 to .81 and accounted for 62% of the explained variance. Table summaries of these results are available on request.

**3.1.2. Scale Reduction.** Due to the two factor structure established in the first EFA it is clear that the SSCCS comprises two separate scales of self-criticism and self-compassion. However, to keep testing time to a minimum we reduced the overall number of response items to three self-compassion and three self-criticism items and reduced the scenarios from eight to five.

The self-compassion scale is composed of four response items, of which “Warm” consistently had the lowest factor loading across all of the scenarios and was therefore removed from the scale. The self-criticism scale is composed of five response items of which “Cold” consistently had the lowest factor loading across all scenarios and was also removed. The next lowest loadings were for “Critical” and “Hostile”, which were very similar, and we decided to remove “Hostile”. Since the self-compassion scale includes the item “Compassionate”, the inclusion of the item “Critical” maintained the comparability of the scales. An additional EFA and internal validity testing was conducted on the reduced items in each scenario. This reaffirmed a two factor structure of self-criticism and self-compassion and showed that the percentage of explained variance increased across the scenarios.

Reducing the number of scenarios was initially determined by the factor loading of each scenario in contributing to the whole scores of the self-compassion scale and the self-criticism scale. Scenario one loaded the least for both the scales and was removed as a result. Scenario two was the second scenario to be removed as this was present in the lowest three scenario loadings in both scales. Scenarios six and seven were also present in the lowest three factor loadings for the self-compassion and self-criticism scales, respectively. We removed scenario six as this had the greatest skew and least variance for the self-compassion scale. EFAs were conducted to establish whether the removal of these three scenarios influenced the factor structure among the remaining scenarios. The scenarios were analysed again with separate summed whole scores from the self-compassion and self-criticism scales. The EFA showed a strong Kaiser-Meyer-Olkin measure of sampling adequacy (.94) and a significant Bartlett’s test of sphericity (*p* < 0.001) for the self-compassion scale across scenarios. The self-criticism scale also had a strong Kaiser-Meyer-Olkin measure of sampling adequacy (.89) and a significant Bartlett’s test of sphericity (*p* < 0.001). The analysis confirmed a one factor structure for each of the scales, with increased percentage of explained variance (Table 1.) Cronbach’s alpha was good for both the self-criticism scale (α = .87) and for the self-compassion scale (α = .91). A complete version of the SSCCS can be found in the appendix.

**3.1.3. Demographic Associations.** There was no significant difference between male scores (M = 33.9, SD = 15.5) and female scores (M = 35.9, SD = 17.6) for the self-compassion scale, *t* (409) = -1.12, *p* = .26. There was also no significant difference found between male scores (M = 53.5, SD = 18.9) and female scores (M = 53.1, SD = 18.6) for the self-criticism scale, *t* (409) = .696, *p* = .86. Linear contrasts showed that age was not associated with either self-criticism or self-compassion scores in our sample (*p* > 0.05).

**3.1.4. Convergent Validity.** Table 2 presents the correlations between the self-compassion and self-criticism scores and the five pre-existing scales of self-criticism and self-compassion. As expected, the self-criticism scores are positively correlated with HS, IS and SC scores, and negatively correlated with RS and SCS scores.

The self-compassion scores did not significantly correlate with HS, IS or SC scores but, as predicted, there were positive correlations with the RS score and with the self-kindness subscale of the SCS (r = 0.21, p < 0.001). Self-compassion did not correlate significantly with the overall SCS (*p* = 0.48).

**4.1 Discussion**

This study demonstrates a clear two-factor structure of the SCCS denoting two separate scales of self-criticism and self-compassion, both of which have excellent internal validity. Furthermore, the study provides evidence for convergent validity with positive correlations between the new self-criticism scale and existing measures of self-criticism, and negative correlations with existing measures of self-compassion and self-reassurance. There were also positive correlations between self-compassion and existing measures of self-reassurance and self-kindness. These correlations indicate that the SCCS is tapping into the desired constructs despite differences introduced by the scenario-based format.

Another finding of this study is that self-criticism and self-compassion did not correlate with one another when measured within the same scenarios. This contrasts with the moderate negative correlations between these constructs on existing measures requiring agreement with more general written statements. These data suggest that the two processes may be independent, and not opposite ends of a bipolar construct, within the context of a specific situation. Thus the format of the SCCS may provide additional flexibility in assessing possible dynamic interactions between self-criticism and self-compassion.

Several questions about the SCCS remain and one of the most important is the extent to which it is sensitive to change. Study two was designed to assess whether the SCCS can adequately measure change in levels of self-compassion and self-criticism in response to negative affect prompted by different types of feedback after a difficult task. Affective responses to feedback are known to depend not just on degree of success and failure but on idiosyncratic causal attributions made for the outcome ([Weiner, 1985](#_ENREF_36)), which are more difficult in the absence of feedback. As lack of feedback creates uncertainty and negative affect in its own right (Epstein, 1972), no prediction was made about whether negative affect would be greater in response to low performance feedback or a no feedback control condition. We hypothesized that state self-compassion and self-criticism would change differentially as a function of negative affect generated by feedback on task performance. Thus, study two was also designed to address the relationship between state affect and SCCS scores, and to confirm that changes in self-criticism and self-compassion could not be reduced to changes in negative and positive affect, respectively.

**5.1. Study Two Method**

**5.1.1. Participants.** A total of 90 participants took part in this study (females 56), none of whom took part in study one. Ninety percent of the participants were aged between 18 – 24 years old and ten percent ranged between 25 – 34 years old. The ethnicity of the sample was varied and included White British (34.4%), White Other (7.8%), Mixed Race (5.6%), Asian British (11.1%), Asian Other (10%), Caribbean (1.1%), African (1.1%), Chinese (28.9%). Experimental procedures were approved by University College London’s Psychology and Language Sciences Ethics Committee.

**5.1.2. Materials and Measurements.** Participants were required to complete a difficult antonym task. This antonym task was developed from the language section of the American Graduate Record Examination. A similar task was implemented by Breines and Chen (2012). The task involves the presentation of a target word and a list of five possible antonyms. The participant has to choose the correct antonym (e.g. “glut”) that corresponds to the target word (e.g. “dearth”). There were 25 trials in total and each trial was randomly presented.

In addition to the antonym task, participants completed the SCCS and two additional mood questionnaires. These mood measures are outlined below:

*The International Positive and Negative Affect Schedule, Short Form (I-PANAS-SF)*

Positive affect (PA) and negative affect (NA) were measured with the 10-item I-PANAS-SF (Thompson, 2007), a cross-culturally reliable and briefer version of the original PANAS (Watson et al., 1988). Participants rated how strongly they were currently experiencing a particular emotion on a 5-point Likert scale (1=*not at all* to 5=*very much so)* (e.g. PA: active, inspired; NA: ashamed, hostile). Cronbach’s alphas for the PA and NA scales are .78 and .76 respectively ([Thompson, 2007](#_ENREF_35)).

*Two Forms of Positive Affect Scale (TFPAS)*

The TFPAS measures the extent to which participants experience 18 different positive emotions ([Gilbert et al., 2008](#_ENREF_13)). Originally used as a trait measure, this scale has been used as a state scale of positive affect ([Rockliff et al., 2011](#_ENREF_32)). Factor analysis revealed three potential forms of positive affect: Active Affect (e.g. energetic, excited), Relaxed Affect (e.g. relaxed, calm) and Safe Affect (e.g. content, warm). The significance of this scale is that it allows for a better approximation of affect systems associated more specifically with self-compassion ([Gilbert, 2010a](#_ENREF_9); [Gilbert et al., 2008](#_ENREF_13)). Participants rate on a 5-point Likert scale (1=*not at all* to 5=*very much so*) how strongly they are experiencing these emotions at the current moment in time. The authors reported a Cronbach’s alpha of .83 for Active and Relaxed Affect, and .73 for Safe Affect scales.

**5.1.3. Procedure.** Participants were initially told that the purpose of the experiment was to investigate the influence of mood on language processing. This was to ensure that any attempts at guessing the hypothesis would not bias participant responses. After providing consent, participants completed the I-PANAS, SCCS and the TFPAS, the order of which was randomized across participants. Participants then completed the antonym task. At the end of the antonym task participants were randomly provided either with feedback in the form of a high or low percentage of correct responses (84% and 28% respectively) or no feedback. No information about normative levels of performance on the test was provided so that participants did not know whether their indicated level of performance was good or bad. They then completed the three questionnaires for a second time. Finally, they were asked to write down what they thought the purpose of the experiment was and the extent to which they found the antonym task difficult (7 point Likert scale where *1= “very difficult”* and *7= “very easy”*).

**5.1.4. Data Processing.** The data were inspected for outliers 2.5 standard deviations above and below the mean. There were no outliers. Scores for the self-compassion scale, the positive and negative PANAS, and the TFPAS sub-scales were not normally distributed and all exhibited a positive skew to varying degrees. For further data analysis these scale scores were log transformed, which normalise their distribution. Data were analysed using 7 separate 2 X 3 mixed-model analyses of variance (ANOVA) with Time (before, after) as the within subjects variable and Feedback (low percentage score, high percentage score, no feedback) as the between subjects variable.

**6.1. Results**

**6.1.1. Difficulty Ratings and Performance.** A one-way ANOVA showed that there was no statistical difference between the three feedback conditions on the perceived difficulty of the antonym task, *F* (2, 87) = 1.23, *p =* .29. The mean score was 2.25 (SD = 1.1) indicating a high degree of perceived difficulty across all three groups. There was also no statistical difference between the three feedback conditions in term of percentage of correct responses, *F* (2, 87) = 1.6, *p =* .207. In addition, performance (mean percentage of correct response = 48.8%, SD = 13.4%) did not correlate with change scores of self-compassion and self-criticism (p > 0.1) for all three groups, indicating that performance was unrelated to changes in self-relating.

**6.1.2. Positive Affect (PANAS).** As shown by the means reported in Table 3, a significant main effect was found for Time, *F*(1,87) = 45, *p <* .001, *η*_­p_^2^ = 0.341, indicating a decrease from time point one to two, but there was no effect of Feedback, *F*(2,87) = 0.35, *p =* .70, *η*_­p_^2^ = 0.008. A significant interaction between Time and Feedback condition was found, *F*(2,87) = 7.9, *p =* .001, *η*_­p_^2^ = 0.155. Post- hoc pairwise comparisons revealed a significant decrease in positive affect scores after no feedback (mean difference = 4.0, *p* < 0.001) and after low percentage score feedback (mean difference = 1.4, *p* = 0.012), but not after high percentage score feedback (mean difference = 0.93, *p* = 0.07). When sex was entered into the ANOVA as a between-subjects variable there were no significant sex effects found (*p* > 0.05).

**6.1.3. Negative Affect (PANAS).** A significant main effect was found for Time, *F*(1,87) = 10.31, *p =* .002, *η*_­p_^2^ = 0.106, indicating an increase in negative affect scores at time point two. There was a significant main effect of Feedback, *F*(2,87) = 3.84, *p =* .025, *η*_­p_^2^ = 0.081, that was qualified by a significant interaction between Time and Feedback, *F*(2,87) = 11.4, *p <* .001, *η*_­p_^2^ = 0.208. Post-hoc pairwise comparisons revealed a significant increase in negative affect scores after no feedback (mean difference = 3.8, *p* = 0.001), which was not the case for low (mean difference = .87, *p* = 0.109) or high (mean difference = .93 , *p* = 0.049) percentage score feedback. When sex was entered into the ANOVA there was a significant main effect, *F*(1,84) = 7.47, *p =* .008, *η*_­p_^2^ = 0.082, revealing a higher score for men (M = 11.2, SD = 4.1) than women (M = 9.5, SD = 3.81).

**6.1.4. Active Affect (TFPAS).** A significant main effect was found for Time, *F*(1,87) = 5.27, *p <* .024, *η*_­p_^2^ = 0.057, indicating an overall decrease, and for Feedback, *F*(2,87) = 4.4, *p =* .015, *η*_­p_^2^ = 0.092, which was qualified by a significant interaction, *F*(2,87) = 3.84, *p <* .025, *η*_­p_^2^ = 0.081. Post-hoc pairwise comparisons revealed a significant decrease in active affect scores after low percentage score feedback (mean difference = 2.33, *p =* 0.008), which was not the case for high score percentage (mean difference = .03, *p =* 0.96) and no feedback (mean difference = .40, *p =* 0.68) conditions. When sex was entered into the ANOVA as a between-subjects variable there were no significant sex effects found (*p* > 0.05).

**6.1.5. Relaxed Affect (TFPAS).** A significant main effect was found for Time, *F*(1,87) = 26.6, *p <* .001, *η*_­p_^2^ = 0.236, indicating a decrease in safe affect scores, and for Feedback, *F*(2,87) = 5.6, *p =* .005, *η*_­p_^2^ = 0.117, qualified by a significant interaction, *F*(2,87) = 15.4, *p <* .001, *η*_­p_^2^ = 0.264. Post-hoc pairwise comparisons revealed a significant decrease in relaxed affect scores after no feedback (mean difference = 5.33, *p* < 0.001), which was not the case for low (mean difference = 1.37, *p =* 0.057) and high (mean difference = .07, *p* = 0.923) percentage score feedback conditions. When sex was entered into the ANOVA as a between-subjects variable there were no significant sex effects found (*p* > 0.05).

**6.1.6. Safe Affect (TFPAS).** A significant main effect was found for Time, *F*(1,87) = 16.5, *p <* .001, *η*_­p_^2^ = 0.163, indicating an overall decrease in safe affect, but not for Feedback, *F*(2,87) = 1.86, *p =* .16, *η*_­p_^2^ = 0.042. There was a significant interaction between Time and Feedback, *F*(2,87) = 8.36, *p <* .001, *η*_­p_^2^ = 0.164. Post-hoc pairwise comparisons revealed a significant decrease in safe affect scores after no feedback (mean difference = 3.1, *p* < 0.001), which was not the case for low (mean difference = .83, *p* = 0.107) and high (mean difference = 1.7, *p* = 0.69) score percentage feedback. When sex was entered into the ANOVA as a between-subjects variable there were no significant sex effects found (*p* > 0.05).

**6.1.7. SCCS Self-Criticism**. A significant main effect was found for Time, F(1,87) = 4.49, p = .037, *η*_­p_^2^ = 0.049, indicating an overall decrease in self-criticism scores, but not for Feedback, F(2,87) = 2.23, p = .113, *η*_­p_^2^ = 0.049. There was a significant interaction between Time and Feedback, F(2,87) = 4.29, p = .017, *η*_­p_^2^ = 0.090. Post-hoc pairwise comparisons revealed a significant reduction in self-criticism scores after no feedback (mean difference = 5.4 , p = 0.005), which was not the case for low (mean difference = .93 , p = 0.54) or high (mean difference = 1.2 , p = 0.36) percentage score feedback (p > 0.05). When sex was entered into the ANOVA as a between-subjects variable there were no significant sex effects found (p > 0.05) with an exception of a three-way interaction between sex, feedback condition and time point, F (2, 84) = 5.96, p = .004, *η*_­p_^2^ = .124.

**6.1.8. SCCS Self-Compassion**. Significant effects were found for Time, F(1,87) = 6.88, p = .010, *η*_­p_^2^ = 0.073, indicating an overall increase, and Feedback, F(2,87) = 3.72, p = .028, *η*_­p_^2^ = 0.079, as well as their interaction, F(2,87) = 5.62, p = .005, *η*_­p_^2^ = 0.115, Post-hoc pairwise comparisons revealed a significant increase in self-compassion scores after no feedback (mean difference = 5.9, p = 0.001), which was not the case for low (mean difference = .77, p = 0.57) or high (mean difference = 1.2 , p = 0.34) percentage score feedback. When sex was entered into the ANOVA as a between-subjects variable there were no significant sex effects found (p > 0.05).

**7.1. General Discussion**

Study two provides preliminary evidence that the SCCS is sensitive to change. The difficult antonym task elicited a reduction in positive affect in the low percentage feedback condition, consistent with the well-established association between outcomes and emotions (Weiner, 1985). The absence of more marked emotional reactions was probably due to the lack of normative information on which to base causal attributions for their performance. Such attributions are the major determinant of achievement-related emotion (Weiner, 1985). More widespread reductions in positive affect and increases in negative affect were observed in the no-feedback condition, consistent with the predictions of Epstein (1972) concerning the effect of uncertainty on anxiety. These were accompanied by compensatory increases in self-compassion and decreases in self-criticism.

This pattern of results, with increased compassion accompanying negative mood changes, is reminiscent of other examples of mood repair processes in the literature (Josephson, Singer, & Salovey, 1996; Power & Brewin, 1990; Sanchez, Vazquez, Gomez, & Joormann, 2014). For example, Power and Brewin (1990) presented participants with the names of hypothetical life events and required them to indicate whether or not a subsequent trait adjective was self-descriptive. In the face of an esteem-threatening life event, participants were slower to endorse negative adjectives and overall endorsed fewer such adjectives as self-descriptive. Josephson et al. (1996) reported that participants low in previous levels of depression were more likely to respond to a sad mood induction by retrieving positive memories on a subsequent autobiographical memory task. Finally, Sanchez et al. (2014) found that participants’ choice to fixate happy rather than sad faces after a negative mood induction predicted mood recovery at the end of the experiment.

The association between negative affect, increased self-compassion, and decreased self-criticism, although consistent with the mood repair literature, is in contrast with results from questionnaire studies, which generally show that higher levels of positive affect are positively correlated with self-compassion and negatively correlated with self-criticism, whereas the opposite is true for negative affect ([Gilbert et al., 2004](#_ENREF_11); [Gilbert & Irons, 2004](#_ENREF_12); [Neff, 2003b](#_ENREF_24); [Neff & Vonk, 2009](#_ENREF_30)). On the surface, these previous studies might lead one to expect increased positive affect in the presence of increased self-compassion and reduced state self-criticism. Questionnaire-based studies cannot, however, capture the dynamic aspects of self-compassion and the way in which it responds to situational determinants. This will necessarily limit the amount of validity that can be obtained from such measures, for example in Study 1. Our data underscore the value of using multiple methods of investigation including the provision of opportunities to repair negative moods. Future research should aim to establish the temporal attributes of the self-compassion process, perhaps with the use of electroencephalography techniques.

**7.1.1 Limitations and Future Directions**

There have been no systematic investigations into whether levels of self-criticism and self-compassion vary as a function of age in adulthood, but there is some evidence to suggest that this may be the case ([Allen, Goldwasser, & Leary, 2011](#_ENREF_1); [Gilbert et al., 2004](#_ENREF_11); [Neff, 2003a](#_ENREF_23); [Neff & Pommier, 2012](#_ENREF_29)). This should be taken into account when using the SCCS, because the majority of our validating samples were young adults.

New studies are emerging that investigate cultural differences in self-criticism and self-compassion ([Ghorbani, Watson, Chen, & Norballa, 2011](#_ENREF_8); [Neff & McGehee, 2010](#_ENREF_27); [Wong & Mak, 2013](#_ENREF_38); [Yamaguchi & Kim, 2013](#_ENREF_39)). Self-compassion has been conceptualised, partially, within the framework of Buddhist psychology ([Neff, 2003a](#_ENREF_23); [Neff, 2003b](#_ENREF_24)), which could suggest increased levels of self-compassion in far-eastern societies. [Neff, Pisitsungkagarn, and Hsieh (2008](#_ENREF_28)) have shown cultural differences between East and West, with increased levels of self-compassion in Thailand as compared to the United States of America. However, they also show variations in self-compassion amongst far-eastern countries, indicating potentially complex interactions between self-compassion and culture. In light of these findings it is important to note that the present study did not assess cultural differences and care should be taken not to overgeneralise the current findings. However, the SCCS does provide an opportunity for future studies to extend the exploration of cultural differences to a situational level of analysis.

There is also evidence in the literature showing sex differences in self-criticism and self-compassion ([Kupeli, Chilcot, Schmidt, Campbell, & Troop, 2013](#_ENREF_19); [Neff, 2003b](#_ENREF_24); [Neff & Pommier, 2012](#_ENREF_29); [Neff & Vonk, 2009](#_ENREF_30)). Our data did not reveal significant differences between male and females. However, there were 95 fewer men than women in our validating sample, which could have influenced these results. Future research should aim to replicate this finding and investigate the potential discrepancies between different questionnaire methodologies as a function of sex.

While the SCCS has not yet been validated within clinical populations it does have potential clinical applications. Firstly, it could be used to investigate similarities and differences between clinical and non-clinical populations in their tendency to respond self-critically or self-compassionately in specific situations. Secondly, the SCCS could be useful to investigate the progress of individuals across therapy sessions. The cultivation of self-compassion through therapy is currently only assessed by changes in general self-statements after several weeks of therapy (Gilbert & Irons, 2004; Gilbert & Procter, 2006; Mayhew & Gilbert, 2008; Neff & Germer, 2012). The SCCS might be useful in predicting the occurrence of self-compassion and self-criticism when patients are faced with specific challenging situations.

**8.1. Acknowledgements.**

This study was supported by the Medical Research Council (Code: MR/J009210/1). We would like to thank Paul Gilbert for his useful comments and his valuable discussions that contributed to the development of the scale.

**References**

Allen, A. B., Goldwasser, E. R., & Leary, M. R. (2011). Self-compassion and Well-being among Older Adults. *Self and Identity, 11*(4), 428-453. Andrews, B. (1995). Bodily shame as a mediator between abusive experiences and depression. *J Abnorm Psychol, 104*(2), 277-285. Andrews, B., Brewin, C. R., Rose, S., & Kirk, M. (2000). Predicting PTSD symptoms in victims of violent crime: The role of shame, anger, and childhood abuse. *J Abnorm Psychol, 109*(1), 69-73.

Blatt, S. J., D'Afflitti, J. P., & Quinlan, D. M. (1979). Depressive experiences questionnaire. *Yale University (Unpublished research manual).*

Brewin, C. R., & Firth-Cozens, J. (1997). Dependency and self-criticism as predictors of depression in young doctors. *J Occup Health Psychol, 2*(3), 242-246.

Brewin, C. R., Firth-Cozens, J., Furnham, A., & McManus, C. (1992). Self-criticism in adulthood and recalled childhood experience. *J Abnorm Psychol, 101*(3), 561-566.

Brewin, C. R., & Shapiro, D. A. (1984). Beyond locus of control: Attribution of responsibility for positive and negative outcomes. *British Journal of Psychology, 75*(1), 43-49.

Ghorbani, N., Watson, P. J., Chen, Z., & Norballa, F. (2011). Self-Compassion in Iranian Muslims: Relationships With Integrative Self-Knowledge, Mental Health, and Religious Orientation. *International Journal for the Psychology of Religion, 22*(2), 106-118.

Gilbert. (2010a). *Compassion Focused Therapy: Distinctive Features*: Taylor & Francis.

Gilbert, P. (2010b). An Introduction to Compassion Focused Therapy in Cognitive Behavior Therapy. *International Journal of Cognitive Therapy, 3*(2), 97-112.

Gilbert, P., Clarke, M., Hempel, S., Miles, J. N., & Irons, C. (2004). Criticizing and reassuring oneself: An exploration of forms, styles and reasons in female students. [Research Support, Non-U.S. Gov't]. *Br J Clin Psychol, 43*(Pt 1), 31-50.

Gilbert, P., & Irons, C. (2004). A pilot exploration of the use of compassionate images in a group of self-critical people. [Research Support, Non-U.S. Gov't]. *Memory, 12*(4), 507-516.

Gilbert, P., McEwan, K., Mitra, R., Franks, L., Richter, A., & Rockliff, H. (2008). Feeling safe and content: A specific affect regulation system? Relationship to depression, anxiety, stress, and self-criticism. *The Journal of Positive Psychology, 3*(3), 182-191.

Gilbert, P., & Procter, S. (2006). Compassionate mind training for people with high shame and self-criticism: overview and pilot study of a group therapy approach. *Clin Psychol Psychother, 13*(6), 353-379.

Hewitt, P. L., & Flett, G. L. (2002). Perfectionism and stress processes in psychopathology. In G. L. F. P. L. Hewitt (Ed.), *Perfectionism: Theory, research, and treatment* (pp. 255-284). Washington, DC, US: American Psychological Association.

Ingram, R. (2003). Origins of Cognitive Vulnerability to Depression. *Cognitive Therapy and Research, 27*(1), 77-88.

Koerner, K., & Linehan, M. M. (1996). Cognitive and interpersonal factors in borderline personality disorder. *Current Opinion in Psychiatry, 9*(2), 133-136.

Koestner, R., Zuroff, D. C., & Powers, T. A. (1991). Family origins of adolescent self-criticism and its continuity into adulthood. *J Abnorm Psychol, 100*(2), 191-197. Kupeli, N., Chilcot, J., Schmidt, U. H., Campbell, I. C., & Troop, N. A. (2013). A confirmatory factor analysis and validation of the forms of self-criticism/reassurance scale. *British Journal of Clinical Psychology, 52*(1), 12-25.

Leary, M. R., Tate, E. B., Adams, C. E., Allen, A. B., & Hancock, J. (2007). Self-compassion and reactions to unpleasant self-relevant events: the implications of treating oneself kindly. *J Pers Soc Psychol, 92*(5), 887-904.

Longe, O., Maratos, F. A., Gilbert, P., Evans, G., Volker, F., Rockliff, H., & Rippon, G. (2010). Having a word with yourself: neural correlates of self-criticism and self-reassurance. *Neuroimage, 49*(2), 1849-1856. Neely, M., Schallert, D., Mohammed, S., Roberts, R., & Chen, Y.-J. (2009). Self-kindness when facing stress: The role of self-compassion, goal regulation, and support in college students’ well-being. *Motivation and Emotion, 33*(1), 88-97.

Neff, K. (2003a). Self-Compassion: An Alternative Conceptualization of a Healthy Attitude Toward Oneself. *Self and Identity, 2*(2), 85-101.

Neff, K. D. (2003b). The Development and Validation of a Scale to Measure Self-Compassion. *Self and Identity, 2*(3), 223-250.

Neff, K. D., & Germer, C. K. (2012). A Pilot Study and Randomized Controlled Trial of the Mindful Self-Compassion Program. *J Clin Psychol*.

Neff, K. D., Hsieh, Y.-P., & Dejitterat, K. (2005). Self-compassion, Achievement Goals, and Coping with Academic Failure. *Self and Identity, 4*(3), 263-287.

Neff, K. D., & McGehee, P. (2010). Self-compassion and Psychological Resilience Among Adolescents and Young Adults. *Self and Identity, 9*(3), 225-240.

Neff, K. D., Pisitsungkagarn, K., & Hsieh, Y. P. (2008). Self-compassion and self-construal in the United States, Thailand, and Taiwan. *Journal of Cross-Cultural Psychology, 39*(3), 267-285.

Neff, K. D., & Pommier, E. (2012). The Relationship between Self-compassion and Other-focused Concern among College Undergraduates, Community Adults, and Practicing Meditators. *Self and Identity, 12*(2), 160-176.

Neff, K. D., & Vonk, R. (2009). Self-compassion versus global self-esteem: two different ways of relating to oneself. *J Pers, 77*(1), 23-50.

Pagura, J., Cox, B. J., Sareen, J., & Enns, M. W. (2006). Childhood adversities associated with self-criticism in a nationally representative sample. *Personality and Individual Differences, 41*(7), 1287-1298.

Rockliff, H., Karl, A., McEwan, K., Gilbert, J., Matos, M., & Gilbert, P. (2011). Effects of intranasal oxytocin on 'compassion focused imagery'. *Emotion, 11*(6), 1388-1396.

Sachs-Ericsson, N., Verona, E., Joiner, T., & Preacher, K. J. (2006). Parental verbal abuse and the mediating role of self-criticism in adult internalizing disorders. *J Affect Disord, 93*(1–3), 71-78.

Southwick, S. M., Yehuda, R., & Giller, E. L. (1995). Psychological dimensions of depression in borderline personality disorder. *Am J Psychiatry, 152*(5), 789-791.

Thompson, E. R. (2007). Development and Validation of an Internationally Reliable Short-Form of the Positive and Negative Affect Schedule (PANAS). *Journal of Cross-Cultural Psychology, 38*(2), 227-242.

Weiner, B. (1985). An attributional theory of achievement motivation and emotion. *American Psychological Association, 92*, 548-573.

Williams, M. J., Dalgleish, T., Karl, A., & Kuyken, W. (2014). Examining the Factor Structures of the Five Facet Mindfulness Questionnaire and the Self-Compassion Scale. *Psychological Assessment*, No Pagination Specified.

Wong, C. C. Y., & Mak, W. W. S. (2013). Differentiating the role of three self-compassion components in buffering cognitive-personality vulnerability to depression among Chinese in Hong Kong. *Journal of Counseling Psychology, 60*(1), 162-169.

Yamaguchi, A., & Kim, M. (2013). Effects of Self-Criticism and Its Relationship with Depression across Cultures. *International Journal of Psychological Studies, 5*(1), 1-10.

**Tables**

**Table 1.** Factor Loadings and Descriptive Statistics for Self-Compassion and Self-Criticism Scales Across Scenarios.

|  |  | Self-Compassion |  |  |  | Self-Criticism |  |
| --- | --- | --- | --- | --- | --- | --- | --- |
| Scenario |  | Loadings |  |  |  | Loadings |  |
|  |  |  |  |  |  |  |  |
| 5. You have just dropped and scratched your new smart phone. |  | 0.86 |  |  |  | 0.82 |  |
|  |  |  |  |  |  |  |  |
| 8. After searching your bag you realize that you have lost a £20 note. |  | 0.86 |  |  |  | 0.82 |  |
|  |  |  |  |  |  |  |  |
| 4. You receive a letter in the post that is an unpaid bill reminder. |  | 0.86 |  |  |  | 0.79 |  |
|  |  |  |  |  |  |  |  |
| 7. You have just opened the washing machine door to find that your white wash has turned pink. |  | 0.85 |  |  |  | 0.81 |  |
|  |  |  |  |  |  |  |  |
| 3. You arrive home to find that you have left your keys at work. |  | 0.83 |  |  |  | 0.81 |  |
|  |  |  |  |  |  |  |  |
|  |  |  |  |  |  |  |  |
|  |  |  |  |  |  |  |  |
| Cronbach's alpha |  | 0.91 |  |  |  | 0.87 |  |
| Mean |  | 35.1 |  |  |  | 53.2 |  |
| SD |  | 16.8 |  |  |  | 18.6 |  |
|  |  |  |  |  |  |  |  |

**Table 2** SCCS Self-Compassion and Self-Criticism Correlations with Existing Measures

|  |  |  |  |  |  |  |  |
| --- | --- | --- | --- | --- | --- | --- | --- |
|  | SCCS Self-Compassion | SCCS Self-Criticism | Self- Criticism^a^ | HS | IS | RS | SCS Self-Kindness^a^ |
|  |  |  |  |  |  |  |  |
| SCCS Self-Compassion | 1 | .026 | -.070 | .011 | -.055 | .132** | .206** |
|  |  |  |  |  |  |  |  |
| SCCS Self-Criticism |  | 1 | .256** | .176** | .299** | -.120* | -.074 |
|  |  |  |  |  |  |  |  |
| Self-Criticism^a^ |  |  | 1 | .522** | .743** | -.433** | -.258** |
|  |  |  |  |  |  |  |  |
| HS |  |  |  | 1 | .543** | -.518** | -.323** |
|  |  |  |  |  |  |  |  |
| IS |  |  |  |  | 1 | -.449** | -.291** |
|  |  |  |  |  |  |  |  |
| RS |  |  |  |  |  | 1 | .603** |
|  |  |  |  |  |  |  |  |
| SCS Self-Kindness^a^ |  |  |  |  |  |  | 1 |
|  |  |  |  |  |  |  |  |
|  |  |  |  |  |  |  |  |
| Mean | 35.1 | 53.2 | 36.4 | 8.8 | 27.8 | 27.5 | 2.9 |
| SD | 16.8 | 18.6 | 11.3 | 4.1 | 7.7 | 5.9 | .76 |
|  |  |  |  |  |  |  |  |
| HS = Hated Self, IS = Inadequate Self, RS = Reassured Self, SCS = Self Compassion Scale. * *P* < .05, ** *p* < .01, ^a^ denotes *N* = 291 |  |  |  |  |  |  |  |

**Table 3.** SSCCS means and (SD) at testing time points for feedback groups.

|  |  |  |  |  |  |  |  |  |
| --- | --- | --- | --- | --- | --- | --- | --- | --- |
|  | High Percentage Score Feedback | |  | Low Percentage Score Feedback | |  | No Feedback | |
|  | Time 1 | Time 2 |  | Time 1 | Time 2 |  | Time 1 | Time 2 |
|  |  |  |  |  |  |  |  |  |
| SCCS Self-Criticism | 58.9 (15.5) | 57.7 (17.1) |  | 57.6 (14.3) | 58.5 (17.2) |  | 53.5 (16.7) | 48.2 (15.5)** |
|  |  |  |  |  |  |  |  |  |
| SCCS Self-Compassion | 33.8 (13.4) | 35.0 (14.5) |  | 30.8 (14.1) | 30.0 (12.8) |  | 37.5 (16.9) | 43.5 (16.1)** |
|  |  |  |  |  |  |  |  |  |
| PANAS positive | 15.0 (4.2) | 14.0 (4.6) |  | 14.2 (3.5) | 12.7 (3.3)** |  | 15.8 (3.3) | 11.8 (3.1) ** |
|  |  |  |  |  |  |  |  |  |
| PANAS negative | 9.6 (4.3) | 8.7 (3.6) |  | 9.7 (4.1) | 10.5 (4.1) |  | 9.6 (4) | 13.4 (3.5)** |
|  |  |  |  |  |  |  |  |  |
| Active Affect | 12.8 (5.1) | 12.8 (5.6) |  | 10.8 (4.6) | 8.4 (5.2)** |  | 12.2 (4.9) | 12.6 (5) |
|  |  |  |  |  |  |  |  |  |
| Relaxed Affect | 14.6 (5.5) | 14.7 (5.9) |  | 13.4 (4.3) | 12.0 (5.5) |  | 13.3 (4.4) | 8.0 (3.8)** |
|  |  |  |  |  |  |  |  |  |
| Safe Affect | 10.5 (3.8) | 10.6 (4.1) |  | 9.5 (3.3) | 8.7 (3.6) |  | 10.0 (3.0) | 6.9 (2.8)** |
|  |  |  |  |  |  |  |  |  |
| ** denotes a significant difference from time point one, *p* < .01. |  |  |  |  |  |  |  |  |

1. The results were also analysed with a principal axis factoring method due to the positive skew of compassionate self-relating. However, the overall factor structure did not differ from the principal components analysis method and factor loadings only differed slightly. [↑](#footnote-ref-1)
