# Supplementary material for: Embodying Compassion: A Virtual Reality Paradigm for Overcoming Excessive Self-Criticism
Source: PLoS One. 2014 Nov 12;9(11):e111933. doi: 10.1371/journal.pone.0111933 (PMC4229123; doi:10.1371/journal.pone.0111933)
Supplement: Information S1 — Methodological Details. (DOCX) [file pone.0111933.s003.docx]

Embodying Compassion: A virtual reality paradigm for overcoming excessive self-criticism

Caroline J. Falconer^1^, Mel Slater^2,3^, Aitor Rovira^2^, John A. King^1^, Paul Gilbert^4^, Angus Antley^2^, & Chris R. Brewin^1*^

**Methodological Details**

# 1. Presence, Body Ownership and Agency - Questionnaires

The ‘VR Experience Questionnaire’ was administered to all participants after they had exited the virtual reality. There were 4 sections concerned with:

A presence in the virtual environment

B the experience in the adult virtual body

C the experience in the child virtual body

D open ended comments.

We adopted the strategy of including many ‘filler’ questions, but there were only three concepts of interest in these questionnaires: presence as the feeling of being in the environment depicted by the displays, body ownership (in both bodies) and agency (the feeling of having controlled the virtual bodies). Questions about body ownership and agency with respect to the child body were not asked for those who had experienced the 3PP condition (since they had not been embodied in any virtual body during that part of the experience).

Table 1 gives the questions important for presence, body ownership and agency, and their medians and Interquartile Ranges.

Table 2 gives the breakdown of those questionnaire variables that were obtained in both the 3PP and 1PP conditions. Recall that 3PP and 1PP refer to the condition only while embodied in the child. Hence, for example, the adult scores for *amedown* refer to the illusion of body ownership in the adult before the child condition had been experienced, since these questions were administered immediately after the adult stage and before the child stage had been experienced. Hence in principle the 1PP and 3PP conditions can have no effect on these scores since those conditions occurred after these scores had been obtained.

Nevertheless there is some evidence from Table 2 that those who were going to experience the 1PP condition tended to give higher body ownership scores than those who were going to go on to experience the 3PP condition. (Of course no participants knew in advance to which condition they had been assigned).

**Table S1** - Medians and Interquartile Ranges for Presence, Body Ownership and Agency Questions in the 1PP condition, n = 22.

Each question was in the form of a statement that the participant could rate on a -3 to 3 scale where -3 = ‘Not at all’, 3 = ‘Very much so’, except for the first question where -3 = ‘...the lab taking part in an experiment’ and 3 = ‘…a room where there was a child crying.

|  |  |  |  |
| --- | --- | --- | --- |
| **Variable** | **Question** | **Median** | **IQR** |
| **Section A: Presence** | | | |
| *There* | I felt like I was in a room where a child was crying as opposed to in a lab taking part in an experiment | 1 | 0,2 |
| **Section B: Body Ownership and Agency as Adult** | | | |
| *amedown* | I felt as if the body I saw when I looked down was my own body. | 1 | 0,2 |
| *amirror1* | I felt as if the body I saw when I looked in the mirror was my own body. | 1 | -1,2 |
| *amirror2* | I had the feeling that I was looking at myself in the mirror rather than looking at someone else. | 0 | -1,2 |
| *atwobodies* | How much did you feel like you had two bodies? (reverse scored) | -1 | -2,1 |
| *aagency* | The movement of the adult’s body responded to the movements of my real body. | 2 | 1,2 |
| **Section C: Body Ownership and Agency as Child** | | |  |
| *cmedown* | I felt as if the body I saw when I looked down was my own body. | 1 | 0,2 |
| *cmirror1* | I felt as if the body I saw when I looked in the mirror was my own body. | 1 | -1,2 |
| *ctwobodies* | How much did you feel like you had two bodies? | 0 | -2,1 |
| *cagency* | The movement of the child’s body responded to the movements of my real body. | 2 | 1,3 |

**Table S2** - Medians and Interquartile Ranges for Presence, Body Ownership and Agency for the Adult condition only by Perspective. z is the two-sample Wilcoxon rank-sum test for testing equal medians, P is the significance level.

|  | **3PP (n=21)** | | **1PP (n=22)** | | |
| --- | --- | --- | --- | --- | --- |
| **Variable** | **Median** | **IQR** | **Median** | **IQR** | **z (P)** |
| *there* | 1 | -1,2 | 2 | 1,2 | -1.51 (0.13) |
| *amedown* | 1 | -1,1 | 2 | 0,3 | -2.25 ( 0.02) |
| *amirror1* | 0 | -1,1 | 1.5 | -1,2 | -1.71 (0.08) |
| *amirror2* | 0 | -1,1 | 1.5 | 0,2 | -2.57 (0.01) |
| *atwobodies* | 0 | -1,0 | -1 | -3,1 | 1.10 (0.27) |
| *aagency* | 2 | 1,2 | 2 | 2,3 | -2.46 (0.01) |
